# The Diagnosis and Treatment of Diseases of Women

**Published:** 1907-11

**Authors:** 


THE DIAGNOSIS AND TREATMENT OF DISEASES OF WOMEN by Harry Sturgeon Crossen, M.D., Clinical Prof. Gynecology, Washington University; Gynecologist to Washington Hospital and Chief of the Gynecological Clinics, etc., etc. C. W. Mosby Medical Book and Pub. Co., St. Louis, Mo.

As stated in the preface this work is devoted exclusively to the Diagnosis and Treatment of Diseases of Women as those diseases are met with in the office and at the bedside by the general practitioner. No space is given to other considerations as etiology, pathology, and major operative technique, except as necessary to bring the work to its highest usefulness as a practical guide along the lines indicated. The important points are given clearly and systematically and make this book an exceedingly useful one for the practitioner, as it deals chiefly with the things he is constantly called upon to do. It is most profusely illustrated with pictures that elucidate the text, there being 700 of them.

